# Central Nervous System Progression/Relapse in Mature T- and NK-Cell Lymphomas

**DOI:** 10.3390/cancers15030925

**Published:** 2023-02-01

**Authors:** Rahul S. Bhansali, Stefan K. Barta

**Affiliations:** Department of Medicine, Division of Hematology and Oncology, Hospital of the University of Pennsylvania, Philadelphia, PA 19104, USA

**Keywords:** T-cell, NK-cell, lymphoma, lymphocyte, relapse, progression, central nervous system, prophylaxis

## Abstract

**Simple Summary:**

Mature T- and NK-cell neoplasms are a heterogeneous group of disease entities that comprise about 15% of non-Hodgkin lymphomas. These subtypes are often aggressive, especially in the relapsed/refractory setting. In particular, central nervous system progression/relapse is a rare but devastating outcome for these patients. Moreover, relative infrequency and heterogeneity of tumor biology have precluded the ability to establish standards of care for prophylaxis and treatment of patients with secondary central nervous system involvement. This review describes the epidemiology and risk factors of central nervous system progression/relapse in patients with mature T- and NK-cell lymphomas and discusses the role of prophylaxis and therapy.

**Abstract:**

Non-Hodgkin lymphomas (NHL) are cancers of mature B-, T-, and NK-cells which display marked biological heterogeneity between different subtypes. Mature T- and NK-cell neoplasms are an often-aggressive subgroup of NHL and make up approximately 15% of all NHL. Long-term follow up studies have demonstrated that patients with relapsed/refractory disease have dismal outcomes; in particular, secondary central nervous system (CNS) involvement is associated with higher mortality, though it remains controversial whether this independently confers worse outcomes or if it simply reflects more aggressive systemic disease. Possible risk factors predictive of CNS involvement, such as an elevated lactate dehydrogenase and more than two sites of extranodal involvement, may suggest the latter, though several studies have suggested that discrete sites of anatomic involvement or tumor histology may be independent risk factors as well. Ultimately, small retrospective case series form the basis of our understanding of this rare but devastating event but have not yet demonstrated a consistent benefit of CNS-directed prophylaxis in preventing this outcome. Nonetheless, ongoing efforts are working to establish the epidemiology of CNS progression/relapse in mature T- and NK-cell lymphomas with the goal of identifying clinicopathologic risk factors, which may potentially help discern which patients may benefit from CNS-directed prophylactic therapy or more aggressive systemic therapy.

## 1. Introduction

Lymphomas are a heterogeneous group of blood cancers with over 70 subtypes defined by the 2022 updates in classifications of lymphoid neoplasms from the World Health Organization [1,2] and International Consensus Classification [3]. These can be broadly subcategorized into mature B-cell and mature T- and NK-cell neoplasms. In this review, we will use the terms mature T- and NK-cell lymphoma (MTNKL) and peripheral T-cell lymphoma (PTCL) interchangeably, unless otherwise specified. PTCL comprises less than 15% of non-Hodgkin lymphomas (NHL) in Western countries, though the prevalence is higher in East Asia. A study by the International T-cell Lymphoma Project observed a 5-year overall survival (OS) rate below 50% for most subtypes of PTCL, with the exception of ALK-positive anaplastic large cell lymphoma (ALK+ ALCL) and subcutaneous panniculitis-like T-cell lymphoma (SPTCL), which were 70% and 64%, respectively [4]. A recent study found that 47% and 21% of patients with PTCL who received first-line therapy were identified as either refractory or relapsed, respectively [5]. Of these patients, the median OS was 5.8 months, further underscoring the aggressive nature of these diseases. Recent advances in the targeted treatment of MTNKL and inclusion of indolent-behaving cutaneous T-cell lymphomas (CTCL) such as mycosis fungoides or primary cutaneous CD30+ T-cell lymphoproliferative disorders likely improve these statistics, though treatment of relapsed and refractory disease remains a major unmet need. 

In particular, central nervous system (CNS) progression/relapse in MTNKL remains poorly defined. This event has been extensively studied in B-cell NHL with an estimated incidence of 1.6 to 12% [6,7,8,9,10,11,12]. It portends a poor prognosis due to several factors including poor CNS penetration of many conventional treatment options, toxicity of chemotherapy with CNS activity, and the symptomatic impact on patient quality of life and fitness. Nonetheless, recent advances in circulating tumor DNA [13] and novel therapies will hopefully improve the outcomes for these patients. Unfortunately, the heterogeneity and relative infrequency of MTNKL have precluded extensive study of CNS progression/relapse in this group of patients. Over the last 10 years, several retrospective studies [14,15,16,17,18,19] have provided valuable insights into the epidemiology and outcomes of CNS progression/relapse in MTNKL and have characterized possible risk factors, though these studies have been limited by small sample sizes and exclusion of certain subtypes, such as CTCL. As such, there is no standard of care defined for the role of CNS-directed prophylactic therapy and subsequent management of CNS progression/relapse in patients with MTNKL. In this review, we describe the epidemiology, risk factors, and outcomes of patients with mature T-cell lymphoma who experience CNS progression/relapse and also discuss the available literature regarding the prevention and treatment of CNS progression/relapse. 

## 2. Brief Overview T-Cell Development and Lymphomagenesis

Defining a cell of origin in MTNKL has historically been difficult, perhaps in part due to the significant heterogeneity of mature T-cells that populate the immune system. Unlike most other hematopoietic precursors which undergo much of their maturation in the bone marrow, T-cells are unique in that their development largely occurs in the thymus. Lympho-myeloid primed precursors and common lymphoid progenitors from the bone marrow migrate to the thymus as thymus-settling precursors (TSPs) (reviewed in [20,21]). TSPs retain the ability to differentiate into multiple lineages, including myeloid cells, B-cells, NK-cells, and dendritic cells [21,22,23,24,25,26,27,28]. However, lymphoid-supporting cytokines and the presence of Notch [29,30,31,32,33,34,35,36,37,38,39,40] and Wnt [41,42,43,44,45] signaling ultimately suppress alternative lineages and drive a transcriptional program leading to T-lineage commitment. It should be noted that some TSPs retain the ability for NK-cell differentiation, though NK-cell development mostly occurs in the bone marrow, as well as the spleen and liver to some extent (reviewed in [46,47]). Intriguingly, while the thymic microenvironment is hospitable for T-cell development, T-cell precursors do not have an intrinsic commitment to this lineage until they reach the double-negative (DN) 2b/3 stage of maturation and undergo T-cell receptor gene rearrangement of β or γδ chains (reviewed in [20,48]). As these cells ultimately mature into naïve αβ- or γδ-lineage T-cells, they enter circulation and can further mature into effector T-cells based on antigenic exposure (reviewed in [49,50]). 

**Figure 1:**
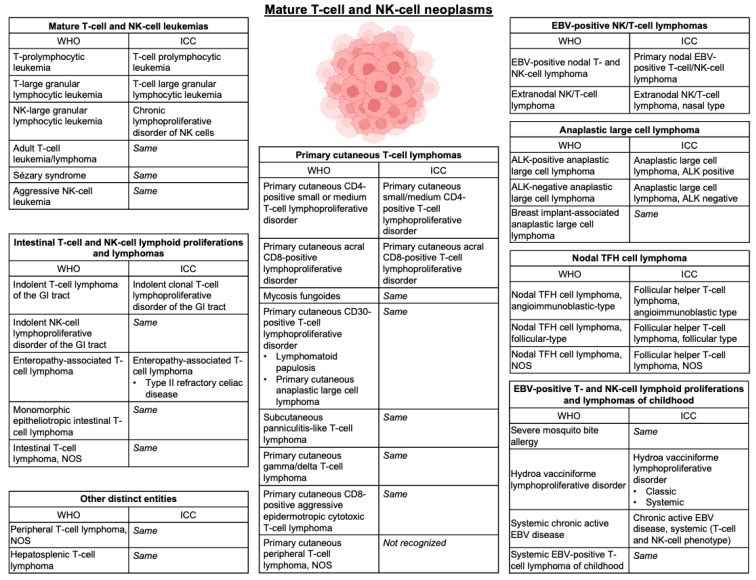
Classification of mature T- and NK-cell lymphomas. Updated classification of mature T- and NK-cell neoplasms depicted in accordance with the World Health Organization (WHO) Classification of Hematolymphoid Tumors, 5th edition [2]. Nomenclature is compared to the International Consensus Classification (ICC) of Mature Lymphoid Neoplasms [3]. Abbreviations—NOS: not otherwise specified; GI: gastrointestinal; EBV: Epstein–Barr Virus; TFH: T-follicular helper; ALK: anaplastic lymphoma kinase.

When post-thymic T-cells develop aberrant proliferation, they can transform into various types of PTCL; these were historically subclassified into nodal, extranodal, cutaneous, and leukemic types based on the predominant tissue affected, though more sophisticated molecular and histologic assays have allowed for an increasingly nuanced categorization based on tumor biology, which often underlies the clinical behavior of these entities (Figure 1) [1,2,3,51]. Certain types of T- and NK-cell lymphomas have stereotypical patterns of extranodal (EN) involvement, such as extranodal NK/T-cell lymphoma (ENKTL) with the paranasal sinuses, enteropathy-associated T-cell lymphoma (EATL) and monomorphic epitheliotropic T-cell lymphoma (MEITL) with the gastrointestinal (GI) lumen, or hepatosplenic T-cell lymphoma (HSTCL) with the liver and bone marrow. Anatomic proclivity can occasionally be explained by chronic, local immunogenic exposure as evidenced by the well-known association of EATL with Celiac disease [52]; additionally, Kern et al. described a subtype of PTCL that expresses CD56, also known as neural cell adhesion molecule (NCAM), which is associated with an increased incidence of CNS (36%), GI (24%), and nasopharyngeal (24%) involvement [53]. Of note, CD56 is highly expressed in ENTKL [54]. Nonetheless, the biological drivers of anatomic tropism remain poorly understood in the majority of MTNKL, particularly regarding CNS involvement. Thus, in the absence of a clear pathobiology to predict which patients may be at highest risk of developing CNS involvement, several groups have aimed to better describe the epidemiology and outcomes of this phenomenon with the goal of ultimately defining risk factors.

## 3. Incidence, Patterns, and Outcomes of CNS Progression/Relapse in MTNKL

### 3.1. Pooled Cohorts

Given the paucity of studies examining CNS progression/relapse exclusively in patients with MTNKL, early data regarding incidence and patient characteristics were from pooled cohorts of patients with aggressive NHL, which are largely B-cell in origin (summarized in Table 1). The Southwest Oncology Group (SWOG) performed a 20-year follow up of the SWOG 8516 study of patients with aggressive NHL in order to better define characteristics of CNS progression/relapse in this population [11]. The study population was comprised of 899 patients enrolled from 1986 to 1991 who had de novo, advanced-stage aggressive NHL. The authors observed an incidence of CNS progression/relapse of 2.8% (*n* = 25), though they did not specify the proportion of patients diagnosed with MTNKL. The median time from initial diagnosis to CNS progression/relapse was 5.4 months with 80% of CNS recurrence occurring during or within 6 months of completion of frontline therapy. Of the 25 patients who developed secondary CNS involvement, 44% (*n* = 11) had isolated CNS relapse and 40% (*n* = 10) had systemic relapse at some point between diagnosis and death. Isolated leptomeningeal disease was observed in 56% (*n* = 14) of patients with CNS disease, while isolated parenchymal disease or mixed involvement occurred in 12% (*n* = 3) and 8% (*n* = 2) of patients, respectively. The remaining patients with CNS progression/relapse either had leptomeningeal disease without evaluation of parenchyma (*n* = 5) or intradural disease (*n* = 1). CNS involvement was associated with a poor outcome, with a median OS of 2.2 months after relapse compared to 9 months for patients without CNS involvement (*p* < 0.0001) and an estimated 2-year OS of 0% and 30%, respectively.

A study from the Norway Radium Hospital reviewed records from 1980 to 1996 in patients over the age of 15 with a diagnosis of NHL (*n* = 2514), which was classified as low-grade, high-grade, lymphoblastic/Burkitt’s, or NHL unspecified [8]. Patients with CNS disease at diagnosis were excluded from study. The authors observed that 4.2% (*n* = 106) of patients developed CNS involvement during first line treatment (*n* = 36) or at relapse (*n* = 70) with a median time to CNS involvement of 5 months and 16 months, respectively. They observed 24.4% (*n* = 20) of patients with lymphoblastic or Burkitt’s lymphoma developed CNS disease compared to only 4.3% (*n* = 52) and 2.8% (*n* = 33) of patients with high-grade and low-grade lymphomas, respectively. Notably, PTCL was considered high-grade though only made up 9.9% (*n* = 121) of this cohort and 4.8% of the entire study population, but the incidence of CNS progression/relapse specifically in patients with PTCL was not reported. Leptomeningeal involvement was seen in 69.8% (*n* = 74) of patients, and parenchymal involvement was seen in 20.8% (*n* = 22) of patients. The median OS was 2.4 and 2.2 months for those who progressed during primary treatment and relapsed, respectively.

Another pooled study from the German High-Grade Non-Hodgkin’s Lymphoma Study Group (DSHNHL) evaluated records from 1693 patients with a diagnosis of an aggressive NHL treated on protocol between 1990 to 2000 [10]. T-cell histology comprised 10% (*n* = 137) of the study population, and patients with CNS disease at diagnosis were excluded. CNS progression/relapse was observed in 2.2% (*n* = 37) of patients, of which only one patient had a mature T-cell lymphoma. B-cell versus T-cell histology was not predictive of risk of CNS relapse at 3 years (2.3% versus 0.9%, *p* = 0.195). The median time from diagnosis to CNS progression/relapse was 4.7 months with parenchymal involvement in 56.8% (*n* = 21), leptomeningeal involvement in 21.6% (*n* = 8), and mixed involvement in 10.8% (*n* = 4) of patients with CNS involvement. Isolated CNS disease was reported in 40.5% (*n* = 15) of patients while 59.5% (*n* = 22) of patients had concurrent systemic progression. The median OS after CNS involvement was 4.4 months with an estimated 3-year OS of 11% compared to 27% in patients with primary progression of non-CNS relapse (*p* = 0.004), respectively; of note, survival data in the T-cell lymphoma subgroup was not specified. After adjusting for other components of the International Prognostication Index (IPI) [55] in a multivariate analysis, the authors found that CNS involvement was an independent risk factor for mortality compared to other sites of relapse and/or primary progressive disease (Relative risk [RR] 2.0, *p* = 0.001)

Ultimately, these pooled analyses consistently demonstrate that CNS progression/relapse occurs early after diagnosis and is associated with poor survival outcomes. Interestingly, the DSHNHL group did not find T- or B-cell histology to be predictive of CNS relapse [10]; however, a minority of patients in these studies had a diagnosis of MTNKL, and only the DSHNHL group clarified the proportion of patients with MTNKL who developed CNS progression/relapse, limiting interpretability in the context of this disease entity.

### 3.2. MTNKL Cohorts

Several groups have also examined MTNKL cohorts to better understand the natural history of disease recurrence and relapse, which have provided further insights into the phenomenon of CNS progression/relapse (summarized in Table 2). López-Guillermo et al. described their experience with 174 patients with PTCL across nine institutions in Spain from 1985 to 1996 [56]. Patients with CTCL were excluded. They reported CNS involvement in 4.6% (*n* = 8) patients, of which one patient had angioimmunoblastic T-cell lymphoma (AITL) and four had angiocentric PTCL, which has since been reclassified as ENKTL; however, this study did not delineate whether patients had CNS involvement at diagnosis or with progression/relapse. Similarly, the British Columbia Cancer Agency evaluated outcomes of 153 patients with PTCL after first relapse and progression [57]. Outcomes were generally poor with a median OS of 5.5 months and median PFS of 3.1 months after relapse or progression. Patients with CNS disease at presentation were excluded from this study, and CNS relapse was observed in 8% of patients (first relapse, *n* = 9; subsequent relapse, *n* = 3). The relative incidence of CNS disease at first relapse/progression based on tumor histology was 5% (*n* = 4) for PTCL, not otherwise specified (NOS), 17% (*n* = 2) for ALK + ALCL, and 8% (*n* = 2) for ALK-negative ALCL (ALK- ALCL).

One of the first major studies specifically examining CNS relapse in a dedicated MTNKL cohort was from a South Korean group who retrospectively analyzed records from 228 patients with MTNKL, though ENKTL and CTCL were excluded [14]. CNS involvement was reported in 8.8% (*n* = 20) of patients during a median follow up time of 13.9 months. Of patients with CNS involvement, PTCL, NOS was the most prevalent subtype (*n* = 11), though based on tumor histology, the incidence of CNS involvement was most common with ALCL (*n* = 5 of 32) followed by EATL (*n* = 1 of 8) and PTCL, NOS (*n* = 11 of 130). Of patients with ALCL and CNS relapse, two were ALK+, two were ALK-, and one was ALK-unspecified. Consistent with data from pooled studies, CNS involvement was an early event with a median time to CNS progression/relapse of 6.1 months from initial diagnosis, though it should be noted that this study included two patients with CNS involvement at initial diagnosis. In 55% (*n* = 11) of patients, CNS relapse occurred during first line or salvage chemotherapy with residual systemic disease; in 35% of patients, CNS relapse occurred after having achieved a complete response to chemotherapy with or without consolidative autologous stem cell transplantation. More patients with CNS involvement had leptomeningeal disease (*n* = 14) than parenchymal (*n* = 5) or mixed disease (*n* = 1). Furthermore, CNS progression/relapse occurred concurrently with systemic involvement in 90% (*n* = 18) of patients. The median OS after CNS relapse was 2.95 months, and the median OS from initial diagnosis was worse compared to those without CNS involvement (7.6 months versus 27.4 months, *p* = 0.009); however, of patients with CNS involvement, only 10% (*n* = 2) died of CNS disease, whereas 55% (*n* = 11) died of systemic disease, perhaps indicating that CNS involvement was more so a reflection of aggressive systemic progression.

The Swedish Lymphoma Registry (SLR) carried out a retrospective analysis of 625 adult patients with MTNKL diagnosed between 2000 and 2009. Of this cohort, 4.5% (*n* = 28) of patients developed CNS progression or relapse at a median time of 4.3 months. Twelve patients relapsed after achieving an initial response to frontline therapy, whereas 15 patients did not respond to initial therapy and experienced CNS progression. PTCL, NOS was the most prevalent subtype with CNS involvement (*n* = 15), and incidence of CNS involvement was highest with PTCL, NOS (6.9%, *n* = 15) and EATL (7.1%, *n* = 4). Consistent with prior studies, leptomeningeal involvement was a more common pattern of disease (*n* = 18) than parenchymal involvement (*n* = 10). Of patients who developed CNS disease at first relapse, isolated CNS involvement was observed in 52% (*n* = 11) of patients, while concurrent systemic involvement was observed in 47% (*n* = 10) of patients. The median OS from relapse or progression was 1.1 months for patients with CNS involvement and 3.8 months for those without CNS involvement (*p* = 0.082). In both groups, about 67% of patients received treatment, and CNS disease did not significantly increase the risk of mortality in univariate (hazard ratio [HR] 1.025, 95% confidence interval [CI] 0.79–1.99, *p* = 0.345) or multivariate (HR 1.6, 95% CI 0.96–2.6, *p* = 0.074) analyses, again suggesting that systemic disease is perhaps the driver of mortality in these patients.

Two North American centers have also published their experiences with CNS relapse in MTNKL. A study from Memorial Sloan Kettering Cancer Center (MSKCC) reviewed an internal database of patients with MTNKL above the age of 15, excluding those with CTCL (except for transformed mycosis fungoides) [17]. Data from 231 patients were reviewed with CNS involvement identified in 6.5% of cases (*n* = 15). CNS progression/relapse was identified early after diagnosis with a median time to CNS involvement of 3.4 months, noting that four patients had CNS involvement at initial diagnosis or prior to first line therapy and three patients developed CNS disease during first line therapy. Of the eight patients who developed CNS relapse after initial chemotherapy, 37.5% (*n* = 3) had isolated CNS relapse while 62.5% (*n* = 5) had concurrent systemic relapse. In patients with CNS involvement, PTCL, NOS was most prevalent (*n* = 6) followed by adult T-cell lymphoma/leukemia (ATLL) (*n* = 4); however, the relative incidence of CNS relapse was most common in ATLL (23.5%, *n* = 4), ENKTL (11.8%, *n* = 2), and HSTCL (11.1%, *n* = 1). The median OS after CNS progression/relapse was 2.6 months. Another study from the University of Texas MD Anderson Cancer Center (MDACC) group evaluated 600 patients with a diagnosis of MTNKL without CNS disease at initial diagnosis [18]. In this cohort, 2.2% (*n* = 13) patients developed CNS progression/relapse at a median time of 6.4 months. Consistent with a short latency to CNS involvement, 62% of patients (*n* = 8) experienced CNS involvement at first progression/relapse, though specific time to CNS progression/relapse was not reported. Notably, 100% (*n* = 13) patients had leptomeningeal involvement with no parenchymal disease observed. PTCL, NOS and ALK + ALCL were the two most prevalent subtypes with CNS relapse (*n* = 4 and *n* = 4, respectively), though the relative incidence of CNS relapse was highest with ALK+ ALCL (5.4%, *n* = 4) and ENKTL (3.7%, *n* = 2). The median OS after CNS progression/relapse in this cohort was 1.5 months with all patients dying except for one patient who was lost to follow up at 2.6 months.

Finally, the Czech Lymphoma Study Group Registry (NiHiL) recently reported the outcomes of patients with MTNKL and CNS relapse [19]. They reviewed cases of patients diagnosed with MTNKL between 1999 and 2020, though excluded those with ATLL, ENKTL, and CTCL. Of 1040 patients evaluated, they observed an incidence of CNS involvement of 2.79% (*n* = 29) with 13 patients demonstrating CNS involvement at initial diagnosis. Median time to CNS progression/relapse was not reported in the other 16 patients. PTCL, NOS was the most common subtype (*n* = 8) in the progression/relapse cohort though the relative incidence was highest amongst patients with ALK + ALCL (10.5%, *n* = 2) and EATL (including MEITL) (3.4%, *n* = 2) who had any type of relapse. Two patients were classified as having primary CNS T-cell lymphoma. Of the 11 other patients with CNS disease at diagnosis, four had parenchymal involvement, four had leptomeningeal involvement, and three had mixed involvement. Conversely, of those who developed CNS disease at progression/relapse, six patients had leptomeningeal involvement, five patients had parenchymal involvement, and five patients had mixed involvement. In the CNS progression/relapse cohort, the median OS after CNS involvement was 11.8 months compared to those 21.3 months with non-CNS relapsed/refractory disease (HR 0.64, 95% CI not reported [NR], *p* = 0.1).

Ultimately, these studies consistently demonstrate CNS progression/relapse as an early event, often occurring within 6 months of initial diagnosis. While some groups, such as the SLR [15] and MDACC [18], attempted to exclude patients with CNS involvement at diagnosis, radiographic and cerebrospinal fluid (CSF) screening were not routinely checked at diagnosis, so early relapses may represent occult involvement already present at diagnosis. CNS involvement occurred in a leptomeningeal pattern in the majority of cases, routinely co-occurred with systemic relapse, and was associated with poor outcomes, as the median OS after CNS involvement was approximately 3 months with the exception of the NiHiL study [19]. However, these reports do not clearly implicate CNS progression/relapse as an independent risk factor for mortality and may rather suggest systemic progression to be the main driver of mortality. However, the retrospective nature, population heterogeneity, and underpowered sample sizes limit the comparative interpretation of these outcomes to patients with non-CNS relapsed/refractory disease.

## 4. Clinicopathologic Risk Factors and Predictive Models

**Figure 2:**
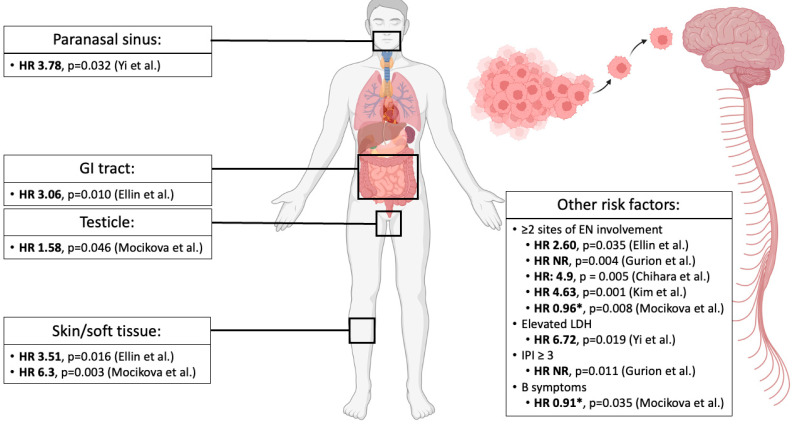
Risk factors for CNS progression/relapse in MTNKL. Several anatomic sites of extranodal involvement have been reported as risk factors for CNS progression/relapse by Yi et al. [14], Ellin et al. [15], and Mocikova et al. [19]. These groups and others [17,18,65] have also found other risk factors predictive of CNS progression/relapse. All HR are reported from multivariate analyses except for Chihara et al. [18], which is reported from a univariate analysis, though the authors state that these data were confirmed in a multivariate model. Abbreviations—HR: hazard ratio; GI: gastrointestinal; EN: extranodal; NR: not reported; LDH: lactate dehydrogenase; IPI: International Prognostication Index. * Hazard ratio values were reported as significant risk factors for CNS progression/relapse by the authors though directionality of ratio was not reported [19].

Several of the aforementioned studies have attempted to identify risk factors associated with CNS progression/relapse in MTNKL (Figure 2), similar to the CNS International Prognostication Index (CNS-IPI) model in large B-cell lymphoma (LBCL), which uses anatomic sites of EN involvement (ENI) in addition to the conventional IPI components (age, Ann Arbor stage, lactate dehydrogenase [LDH], performance status, number of sites of ENI) to predict the risk of CNS relapse [12]. Yi et al. examined various baseline clinical characteristics as well as components of the IPI to assess risk factors for CNS progression/relapse in 228 patients with MTNKL, excluding ENKTL and primary CTCL [14]. Twenty CNS events were observed. Of the standard IPI parameters, only a LDH above the upper limit of normal (ULN) was associated with an increased risk of CNS progression/relapse in both univariate (RR 7.28, 95% CI 1.47–36.03, *p* = 0.015) and multivariate (HR 6.72, 95% CI 1.55–29.13, *p* = 0.011) analyses. Histologic subtypes were not included in this risk modeling, though paranasal sinus involvement was also a risk factor for CNS progression/relapse in both univariate (RR 3.51, 95% CI 1.13–10.89, *p* = 0.029) and multivariate (HR 3.78, 95% CI 1.42–10.08, *p* = 0.008) analyses. Other risk factors which have been proposed in B-cell lymphomas, such as bone marrow involvement, visceral organ involvement, or the composite IPI score (reviewed in [58]) did not confer a significantly increased risk of CNS involvement. In comparing patients from their cohort with zero, one, or two risk factors (defined as LDH above the ULN and/or paranasal sinus involvement), they observed a cumulative incidence of CNS involvement of 1.3%, 10.6%, and 23.8%, respectively (*p* = 0.01), supporting the predictive nature of these factors.

The SLR study [15] assessed similar components as possible risk factors for CNS involvement in a larger cohort of 625 patients with MNKTL with an incidence of CNS progression/relapse of 4.5% (*n* = 28). Similar to the South Korean group [14], the anatomy of ENI appeared to be predictive of CNS relapse. In both univariate and multivariate analyses, there was a significant association between skin involvement (univariate: HR 4.33, 95% CI 1.75–10.7, *p* = 0.002; multivariate: HR 3.51, 95% CI 1.26–9.74, *p* = 0.016) and GI involvement (univariate: HR 2.90, 95% CI 1.28–6.60, *p* = 0.011; multivariate: HR 3.06, 95% CI 1.30–7.18, *p* = 0.010) with CNS progression/relapse. Additionally, ≥2 sites of ENI was also a risk factor for CNS progression/relapse in univariate (HR 4.05, 95% CI 1.83–8.98, *p* = 0.001) and multivariate (HR 2.60, 95% CI 1.07–6.29, *p* = 0.035) models. Moreover, the presence of at least one of these identified risk factors was associated with an increased risk of CNS progression/relapse in their cohort using the Kaplan–Meier method (HR 3.2, 95% CI 1.52–6.69, *p* = 0.002). Sinus involvement was not specifically assessed as an anatomic risk factor, and LDH above the ULN, IPI score, or histologic subtype were not found to be significant risk factors in a univariate model. When the authors performed this same risk modeling in patients treated with CHOP (cyclophosphamide, doxorubicin, vincristine, and prednisone), with CHOEP (cyclophosphamide, doxorubicin, vincristine, etoposide, and prednisone), and for patients with CNS involvement only in first relapse/progression, skin involvement, GI involvement, and ≥2 sites of ENI remained significant risk factors.

In the MSKCC study of 231 patients, Gurion et al. [17] included patients with ENKTL and ATLL, which were excluded from the South Korean [14] and SLR [15] studies, respectively. CNS progression/relapse occurred in 7% (*n* = 17) of patients. Indeed, tumor histology in this cohort did appear to have some predictive value for CNS relapse, as patients with ATLL had a significantly higher risk of CNS involvement compared to all other histologies (HR NR, *p* = 0.001) in a univariate analysis. Additional risk factors in univariate modeling were advanced stage (HR NR, *p* = 0.033), bone marrow involvement (HR NR, *p* = 0.022), ≥2 sites of ENI (HR NR, *p* < 0.001), and IPI score ≥3 (HR NR, *p* < 0.01). In a multivariate Cox regression, only ≥2 sites of ENI (HR NR, *p* = 0.004), ATLL histology (HR NR, *p* = 0.009), and high IPI score (HR NR, *p* = 0.011) were retained as significant risk factors for CNS progression/relapse. Given that ≥2 sites of ENI is a component of the IPI, the authors confirmed that this independently predicted CNS progression/relapse within patients with a high IPI score (*p* = 0.047). In regard to the predictive significance of ATLL, these findings are in line with the well-established risk of CNS involvement in this population [59]. The MDACC group [18] analyzed 600 patients with MNKTL and included tumor histology in their assessment of risk factors for CNS relapse, though excluded ATLL and CTCL. The incidence of NCS progression/relapse was only 2.2% (*n* = 17) in this cohort. Although there was a relative increase in incidence among patients with ALK+ ALCL, this did not meet the threshold for statistical significance (HR 8.2, 95% CI 0.9–73.5, *p* = 0.060) when compared to AITL, though this could be in part due to the small event rate in this cohort. No other patient characteristics were significantly associated with CNS progression/relapse with the exception of ≥2 sites of ENI, which again demonstrated an increased risk of this event with univariate (HR 4.9, 95% CI 1.6–15.0, *p* = 0.005) and multivariate analysis (data NR).

The NiHiL study [19] performed a subgroup analysis of patients with CNS involvement at relapse/progression and found that they were more likely to have advanced stage (*p* = 0.04), soft tissue involvement (*p* = 0.005), testicular involvement (0.008), ≥2 sites of ENI (*p* = 0.019), and a high IPI score (*p* = 0.039) compared to patients with non-CNS relapse. Using a multivariate model, soft tissue (HR 9.3, 95% CI NR, *p* = 0.003) and testicular (HR 1.58, 95% CI NR, *p* = 0.046) involvement were found to be risk factors for CNS involvement, while bone marrow, GI, and skin involvement were not. Of note, B symptoms (HR 0.91, 95% CI NR, *p* = 0.035) and ≥2 sites of ENI (HR 0.96, 95% CI NR, *p* = 0.003) were both reported as independent risk factors for CNS involvement, though the directionality of the HR comparison was not reported.

In multivariate modeling, ≥2 sites of ENI is commonly observed to be a risk factor for CNS relapse in MTNKL, while an elevated LDH, high IPI score, and B symptoms are inconsistently reported as significant risk factors. While ≥2 sites of ENI could reflect increased tumor burden, the lack of these other risk factors does not convincingly support tumor burden as an independent driver of CNS progression/relapse. Several of these studies suggest a role for the anatomic site of ENI in predicting CNS involvement, although these sites vary study-to-study; nonetheless, this may implicate an aspect of the tumor biology which renders it more prone to seeding the CNS, though this has yet to be established in preclinical or clinical models. Interpretation of these results, however, is limited by the small sample size and low number of events in each separate cohort.

### ATLL and ENKTL

As many studies have inconsistent inclusion of ATLL and ENKTL, these entities require special attention since they have historically been associated with an increased risk of CNS involvement. As above, the MSKCC group observed ATLL histology to be a risk factor for CNS progression/relapse [17]. Due to its association with chronic human T-lymphotropic virus type 1 (HTLV-1), ATLL is consequently predominant in regions with high HTLV-1 prevalence, such as Japan, the Caribbean, and South America [60]. In addition, the biology of this tumor may vary with geography [61], so this patient population has been excluded from most of these international studies regarding CNS relapse. However, the first case series describing CNS involvement in ATLL was reported over 30 years ago from a Japanese group [59]. In 99 patients with ATLL, Teshima et al. describe an incidence of CNS involvement of 10.1% (*n* = 10) with one patient having CNS involvement at initial diagnosis. ATLL has several subtypes—smoldering, chronic, acute (leukemic), and lymphomatous—that vary in prognosis, though CNS involvement was more commonly seen in the lymphomatous subtype (*n* = 9). Consistent with other studies of PTCL, most patients (*n* = 6) had leptomeningeal involvement, and time to CNS progression/relapse was short after diagnosis (3.8 months). Moreover, there was 100% 1-year mortality with a median OS of 5.2 months after CNS progression/relapse. Interestingly, and perhaps reflective of the aggressive nature of acute and lymphomatous ATLL, 80% (*n* = 8) of patients with CNS involvement died of systemic progression, and the survival of patients with acute or lymphomatous ATLL and CNS involvement (*n* = 9) versus those without CNS involvement (*n* = 56) did not differ significantly (*p* > 0.05). Another study presented at the American Society of Hematology (ASH) Annual Meeting and Exposition in 2021 specifically evaluated CNS progression/relapse in North American patients with ATLL, given that this has a distinct biologic signature; of 65 patients with ATLL, they noted an incidence of 30.8% (*n* = 20) of CNS involvement [62]. They did not find clinical aspects of ATLL, such as hypercalcemia, elevated LDH, or leukocytosis/lymphocytosis, to be predictors of CNS involvement; however, similar to the Japanese cohort, acute/lymphomatous variants were strongly associated with CNS involvement (*p* = 0.00002). In regard to predicting mortality in patients with ATLL and CNS involvement, only bone marrow involvement was associated with worse survival compared to those without CNS disease (*p* = 0.038). Ultimately, these data reflect that patients with acute or lymphomatous ATLL are the most likely to develop CNS relapse regardless of geographic variations in disease biology.

ENKTL is another MTNKL subtype with reported CNS involvement, though with a lower frequency than ATLL; a study from Kim et al. [63] reported an incidence of CNS progression/relapse in 5.8% of patients with ENKTL (*n* = 12) with a median time from diagnosis of 11.6 months, which is longer than reported other subtypes of lymphoma. Half of the patients (*n* = 6) had leptomeningeal involvement, while the other half (*n* = 6) had parenchymal involvement. Consistent with other forms of MTNKL, the median OS after CNS involvement was short (2.53 months). The risk of CNS involvement in these patients may partially stem from the invasive nature of ENTKL and the proximity of the nasopharynx and paranasal sinuses to the CNS, which would be consistent with the findings by Yi et al. [14] even though their study excluded patients with ENKTL. However, Kim et al. [63] observed that patients with ENKTL without upper aerodigestive involvement were at a higher risk of CNS involvement compared to those with upper aerodigestive disease (RR 4.7, 95% CI 1.5–14.5, *p* = 0.008) in a univariate analysis. Other risk factors identified were lymph node involvement (RR 8.4, 95% CI 1.8–38.5, *p* = 0.006), advanced stage disease (RR 17.7, 95% CI 3.9–81.6, *p* < 0.001), and advanced NK/T-cell lymphoma prognostic index (NKPI) risk group (RR 10.4, 95% CI 2.2–48.4, *p* = 0.003). As the NKPI scoring system is comprised of B symptoms, elevated LDH, advanced stage, and lymph node involvement [64], they performed a multivariate analysis with the NKPI score, primary site of involvement, and IPI score and found that the NKPI score was retained as an independent prognostic factor (RR 9.3, 95% CI1.8–47.2, *p* = 0.007). The same group published an updated report in order to create a predictive model for CNS progression/relapse in patients with ENKTL called the CNS-prognostic index of natural killer (CNS-PINK) model [65]. They had a training cohort of 399 patients with ENTKL, of which 6.8% (*n* = 27) developed CNS progression/relapse. Of patients with CNS disease, 11 had leptomeningeal disease, 10 had parenchymal disease, and six had mixed involvement. The median time to CNS involvement was 10.1 months and the median OS after CNS progression/relapse was 3.7 months, which are similar to their prior study [63]. Of note, patients with CNS progression/relapse had a significantly shorter overall survival compared to those without CNS involvement (15.1 months versus 98.9 months, *p* < 0.001). In a univariate analysis, the authors observed an increased risk of CNS involvement in patients with an elevated LDH (HR 2.8, 95% CI 1.3–6.0, *p* = 0.01), EBV positivity (HR 3.2, 95% CI 1.3–8.0, *p* = 0.013), ≥2 sites of ENI (HR 7.1, 95% CI 3.2–15.6, *p* = 0.001), distant nodal involvement (HR 4.4, 95% CI 2.0–9.5, *p* = 0.001), advanced stage (HR 6.7, 95% CI 3.0–15.0, *p* = 0.001), and high (HR 7.3, 95% CI 2.6–20.8, *p* = 0.001) or intermediate/high prognostic index of natural killer lymphoma (PINK) score (HR 5.1, 95% CI 1.9–13.4, *p* = 0.001); the PINK score consists of an age over 60 years, advanced stage, distant nodal involvement, and non-nasal type [66]. However, in a multivariate model, the only remaining risk factor was ≥2 sites of ENI (HR 4.6, 95% CI 2.0–10.9, *p* = 0.001). A high/intermediate PINK score seemed to correlate with CNS relapse, though did not meet the threshold for statistical significance (HR 2.7, 95% CI 0.9–7.7, *p* = 0.066), though the authors combined these two risk factors to develop the CNS-PINK model. The 2-year cumulative incidence of CNS involvement was 4.1% and 22.8% for patients with a CNS-PINK score of 0–1 (low risk) or 2 (high risk). These results were corroborated in a validation cohort of 253 patients from the Japanese Next-Generation Therapy for NK/T-Cell Lymphoma in East Asia dataset [67], with a cumulative incidence of CNS involvement of 4.5% and 13.9% for low risk and high risk groups, respectively (*p* = 0.038).

## 5. Role of CNS Prophylaxis and Frontline Therapy

Despite substantial efforts to identify patients at risk of CNS progression/relapse, the role of prophylactic therapy remains controversial. Recently, a large multi-institutional, real-world study in aggressive B-cell NHL (excluding Burkitt lymphoma and transformed chronic lymphocytic leukemia) assessed the use of intrathecal (IT) or systemic methotrexate [MTX] or cytarabine [AraC]) CNS-directed prophylaxis in preventing CNS progression/relapse [68]. Of 1162 patients evaluated, 77% (*n* = 894) received IT therapy and 20% (*n* = 236) received HD-MTX, though the rate of CNS involvement was similar in both groups (5.4% versus 6.8%, respectively) and observed CNS progression/relapse was consistent with expected CNS progression/relapse rates based on the CNS-IPI (5.8% versus 5.7%), ultimately questioning the efficacy of CNS prophylaxis even in high-risk patients.

The role of chemoprophylaxis has been less extensively studied in MTNKL, and the 2022 National Comprehensive Cancer Network (NCCN) guidelines for T-cell lymphomas [69] do not routinely recommend CNS evaluation unless a patient displays symptoms. An exception is for those with ATLL, in whom the NCCN recommends radiographic and/or CNS evaluation in any patient with acute or lymphomatous subtype along with CNS-directed prophylaxis. These recommendations are in line with those from the Revised ATLL International Consensus Meeting Report [70]. This rationale stems from the previously discussed incidence of CNS progression/relapse in this population. An early study by the Japan Clinical Oncology Group (JCOG), known as JCOG9109, examined induction regimens with deoxycoformycin in ATLL and did not include CNS prophylaxis [71]. The overall outcomes were poor in these patients with a median OS of 7.4 months and an estimated 2-year OS of 15.5%; however, the incidence of CNS progression/relapse was only 1.6%, though this may be underestimated since CSF was not routinely evaluated, and many patients may have succumbed to systemic disease prior to the onset of neurologic symptoms. Nonetheless, with increasing evidence of CNS involvement with ATLL, other JCOG studies (JCOG9303 [72] and JCOG9801 [73]) incorporated CNS prophylaxis with IT MTX/prednisone and IT MTX/IT AraC/prednisone, respectively, into a systemic backbone of VCAP (vincristine, cyclophosphamide, doxorubicin, and prednisone), AMP (doxorubicin, ranimustine, and prednisone) and VECP (vindesine, etoposide, carboplatin, and prednisone). An additional caveat of this regimen is that both etoposide and ranimustine are able to penetrate the blood–brain barrier to some degree [74]. Moreover, JCOG9303 assessed VCAP-AMP-VECP as a single-arm study, while JCOG9801 was a randomized control trial comparing it to a biweekly CHOP with IT prophylaxis, and patients with CNS involvement at diagnosis were excluded from study. CNS involvement was observed in 6.3% of patients in JCOG9303 and 3.5% of patients in the VCAP-AMP-VECP arm in JCOG9801 (compared to 8.2% with CHOP), suggesting some possible benefit to CNS-directed therapy in ATLL, though this has yet to be examined prospectively. In addition to IT therapy, anecdotal experiences also support the use of intercalating high-dose MTX (at least 3 g/m^2^) with induction, etoposide-containing induction regimens, systemic MTX/AraC-containing induction regimens, and thiotepa-based conditioning regimens for patients undergoing consolidative stem cell transplants, all of which theoretically offer some degree of CNS protection [75].

Unfortunately, the data supporting the use of CNS-directed prophylactic therapy are even more sparse outside of ATLL. Based on their robust risk stratification in ENKTL, Kim et al. had proposed using a scoring system for this disease to identify patients in whom CNS prophylaxis may be appropriate [63]; in their later CNS-PINK study [65], they retrospectively compared patients in the training cohort whose chemotherapy regimen included intermediate-dose MTX (at least 2 g/m^2^) or not and found no significant difference in the cumulative incidence of CNS progression (5.0% and 7.4%, respectively). However, when using a risk stratification based on the CNS-PINK score, the authors found that patients with high risk had a significantly lower incidence of CNS progression/relapse if they received intermediate dose MTX (*p* = 0.029), though there was no significant difference in the low-risk group (*p* = 0.43). These findings were not replicated in the validation cohort, though the sample size was likely underpowered. Moreover, limitations of this subgroup analysis include the confounding effect of regimens containing CNS-penetrating chemotherapy (etoposide and ifosfamide) as well as the evaluation of the utility of intermediate-dose of MTX, as it is commonly accepted that at least 3 g/m^2^ (high-dose) is needed for adequate CNS activity. Moreover, even high-dose MTX has not consistently been demonstrated to offer a protective effect from CNS progression/relapse in LBCL, including high-risk subtypes [68,76,77]. Nonetheless, the benefit seen in the high-risk group of their training cohort is certainly provocative and warrants prospective evaluation.

More broadly, the previously discussed retrospective case series have not demonstrated a benefit to CNS-directed prophylaxis in the overall population of patients with MTNKL. No patients in the South Korean group study had received CNS-directed therapy prior to CNS involvement [14]. Additionally, the MDACC group did not routinely offer CNS prophylaxis and had insufficient data on patients who received prophylactic therapy to assess its impact on CNS progression/relapse, though they noted that induction regimens containing systemic MTX and AraC (i.e., HyperCVAD/MA [hyperfractionated cyclophosphamide, vincristine, doxorubicin, dexamethasone alternating with systemic MTX and AraC) did not significantly decrease the incidence of CNS progression/relapse [18]. The SLR group observed that 5.9% (*n* = 3) of patients who received IT prophylaxis developed CNS progression/relapse, and the risk of CNS involvement was not mitigated by either IT prophylaxis (HR 1.3, *p* = 0.7), use of an etoposide-containing regimen (HR 1.1, *p* = 0.8), or early autologous stem cell transplantation (HR 0.7, *p* = 0.4) [15]. Gurion et al. observed that 10.3% (*n* = 24) of patients in the MSKCC cohort received CNS prophylaxis, most commonly IT MTX (*n* = 21) [17]. CNS prophylaxis was used due to HTLV-1 positivity (*n* = 8), ≥2 sites of ENI (*n* = 5), bone marrow involvement (*n* = 3), high IPI score (*n* = 3), and for testicular involvement (*n* = 1). However, 12.5% (*n* = 3) of these patients still developed CNS progression/relapse compared with 5.9% (*n* = 12) of those who did not receive any CNS prophylaxis (*p* = 0.194). Even when stratified by IPI score, HTLV-1 status, or LDH, CNS prophylaxis did not appear to significantly reduce the risk of CNS progression/relapse. In the NiHiL study, five patients received HyperCVAD/MA, none of whom had CNS progression/relapse [19]. Prophylaxis with IT MTX was specifically offered to those with testicular involvement (*n* = 4), with one of these patients still having a leptomeningeal relapse. CNS progression/relapse rates were similar between patients who received CHOP versus CHOEP (3.8% versus 2.6%). However, the small sample sizes of these groups preclude any definitive analysis regarding efficacy.

Ultimately, there have been no prospective studies comparing the role of CNS-directed prophylaxis in patients with MTNKL. While some data may suggest a possible benefit to certain patients with a high-risk histology, such as acute/lymphomatous ATLL or ENKTL with a high-risk CNS-PINK score, these studies are limited by the small sample sizes and retrospective nature. Moreover, the overall rarity of CNS progression/relapse in MTNKL, which itself is a rare disease, will likely present an ongoing barrier in addressing this question as studies are likely to be statistically underpowered to detect differences in these infrequent events. Of note, a recent presentation at the ASH Annual Meeting and Exposition in 2021 in a cohort of patients with MTNKL with CNS progression/relapse (*n* = 83) found that the use of CNS prophylaxis (*n* = 17) did not significantly impact the time to CNS involvement (HR 1.4, 95% CI 0.7–2.6, *p* = 0.18) [78].

## 6. Treatment of CNS Progression/Relapse

As alluded to above, the outcomes of patients with CNS progression/relapse are poor, with the median OS after CNS involvement ranging from 1.1 to 11.8 months, with only the study by Mocikova et al. reporting a median OS over 6 months [14,15,16,17,18,19,78]. This likely stems from a concurrence with systemic progression, underscoring an aggressive disease phenotype. There is currently no standard of care for these patients. In their cohort of 20 patients, Yi et al. reported the use of radiation (RT), IT therapy, and/or systemic therapy in eight, nine, and eight patients, respectively [14]. Eight patients had a combination of at least two of these therapies. A complete response in the CNS was observed in 45% (*n* = 9) of patients, and three patients underwent subsequent autologous stem cell transplant, with one of them surviving an additional 8.3 years. In the MDACC cohort, four patients received best supportive care with steroids, one patient received RT, and eight patients received salvage chemotherapy with or without concomitant IT therapy [18]. One patient underwent consolidative allogeneic stem cell transplant after achieving a complete response but died due to treatment complications. Specific outcomes based on treatment modalities were not reported. The Czech group noted that eight patients with CNS involvement at relapse received high-dose MTX, often in combination with other agents including AraC or thiotepa [19]. Intrathecal treatment was infrequently used in the relapsed setting, though several salvage approaches were used, including platinum-based therapy (*n* = 5), whole brain RT (*n* = 5), and brentuximab vedotin in combination with gemcitabine (*n* = 1). Consolidative therapy after CNS progression/relapse was performed with autologous stem cell transplantation in five patients, one of whom underwent subsequent allogeneic stem cell transplantation. Conditioning regimens consisted of carmustine, etoposide, AraC, and melphalan (BEAM, *n* = 2); thiotepa, etoposide, AraC, and melphalan (TEAM, *n* = 2); and thiotepa + carmustine (*n* = 1). Of note, two patients who underwent autologous stem cell transplantation had previously undergone whole brain RT. Of the 16 patients who were treated for CNS progression/relapse, only two patients achieved a complete response (one after eventual allogeneic stem cell transplantation and one after whole brain RT + autologous stem cell transplantation). In the study by Bhansali et al., there was no significant difference in the median OS after CNS progression/relapse in patients who received salvage IT therapy, salvage systemic therapy, or both, whether or not the patients had isolated CNS (5.8 months, 7.2 months, and 4.8 months, respectively) or combined CNS and systemic relapse (3.7 months, 6.3 months, and 2.5 months, respectively) [78].

While the survival outcomes of patients with CNS progression/relapse in these studies is dismal, it should be emphasized that many of these are retrospective in nature and the cases described often precede the modern era of novel agents in MTNKL, such as romidepsin [79,80], belinostat [81], brentuximab vedotin [82,83,84], lenalidomide [85], and duvelisib [86]. While patients with CNS disease were excluded from many of the clinical trials leading to the approval of these drugs [79,80,81,83,84,86], case reports suggest the possible efficacy of these agents in patients with CNS disease [87,88,89]. Furthermore, data on the efficacy of agents such as lenalidomide in patients with B-cell NHL with primary or secondary CNS involvement [90,91] are encouraging and may potentially be extrapolated to MTNKL, though this warrants more formal investigation. Consequently, as these agents become increasingly incorporated into salvage regimens for relapsed/refractory disease, hopefully we will gain more insights into their activity against CNS disease.

## 7. Conclusions

Mature T- and NK-cell lymphomas are a difficult-to-treat group of diseases due to their low prevalence, aggressive nature, and heterogeneous biology. While uncommon, CNS progression/relapse occurs at a similar frequency as in LBCL and is associated with dismal outcomes. Several case series over the last decade have provided insights into the epidemiology of this event with consistent evidence that it occurs early after initial diagnosis and is often associated with systemic relapse.

Given the relative infrequency of CNS progression/relapse in MTNKL, a clear biological or molecular mechanism has not yet been elucidated. As previously discussed, patients whose tumors harbor NCAM (CD56) expression appear to have higher risk of CNS involvement along with GI and nasopharyngeal involvement [53], which are notably considered possible high-risk anatomic sites for CNS progression/relapse [14,15]. Molecular profiling of LBCL has identified *MYD88* mutations as a possible risk factor for CNS involvement [92], though there was no enrichment for alterations of JAK/STAT signaling or epigenetic pathways, which are more common in patients with MTNKL. Immune evasion may also contribute to CNS involvement, which can be driven by subtype-specific alterations in PD1/PD-L1 expression [93,94,95], apoptotic dysregulation [96,97,98,99], or an immunosuppressive tumor microenvironment [100]. Ultimately, CNS progression/relapse in MNKTL remains poorly understood mechanistically, though prospective studies are warranted and should utilize advances in molecular profiling to better define patients at highest risk.

Regarding the prevention of CNS involvement, there has not been any compelling evidence to support the use of CNS-directed prophylactic therapy in patients with MTNKL with the exception of acute/lymphomatous ATLL. Moreover, having ≥2 sites of ENI appears to be a consistent risk factor for CNS progression/relapse, so prophylactic therapy could be considered in this case, particularly if the patient has a higher-risk histology such as ENTKL. Treatment of CNS progression/relapse remains a major unmet need in patients with MTNKL; while high-dose MTX may be beneficial in achieving a CNS response, a major driver of mortality in these patients is concomitant systemic disease. However, the increasing prevalence and development of novel agents may offer a hopeful future for patients.

## Figures and Tables

**Table 1:** Summary of pooled studies describing incidence, patterns, and outcomes of CNS relapse in patients with MTNKL.

Pooled Study *	Total Population Evaluated, *n*	Patients with MTNKL, *n* (%)	Patients with CNS Relapse/Progression, *n* (%)	Pattern of CNS Involvement, *n* (%)	Pattern of Relapse, *n* (%)	Median TTCNS, Months ^†^	Median OS, Months ^‡^	Comments
Bernstein et al. [11]	899	NR	25 (2.8)	Leptomeningeal—14 (56) Parenchymal—3 (12) Mixed—2 (8)	Isolated CNS—11 (44) CNS + systemic—10 (40)	5.4	2.2	
Hollender et al. [8]	2514	121 (4.8)	222 (8.8)	Leptomeningeal—74 [69.8] Parenchymal—22 (20.8)	NR	5 ^a^ 16 ^b^	2.4 ^a^ 2.2 ^b^	^a^ Patients who progressed during first line treatment ^b^ Patients who relapsed after first line treatment
Boehme et al. [10]	1693	137 (8.1)	37 (2.2)	Leptomeningeal—8 (21.6) Parenchymal—21 (56.8) Mixed—4 (10.8)	Isolated CNS—15 (40.5) CNS + systemic—22 (59.5)	4.7	4.4	Of patients with CNS relapse, only 1 had MTNKL

* Data are from pooled cohort and do not specify MTNKL subgroup unless otherwise specified. ^†^ Median time from initial diagnosis to CNS relapse and/or progression. ^‡^ Median overall survival after CNS relapse. NR: Not reported.

**Table 2:** Summary of MTNKL-specific studies describing incidence, patterns, and outcomes of CNS relapse in patients with MTNKL.

MTNKL Study	Total Population Evaluated, *n* ^§^	Patients with CNS Relapse/Progression, *n* (%)	Histology-CNS, *n* (%) ^#^	Histology-Total, *n* (%) ^$^	Pattern of CNS Involvement, *n* (%)	Pattern of Relapse, *n* (%)	Median TTCNS, Months ^†^	Median OS, Months ^‡^	Comments
López-Guillermo et al. [56]	174	8 (5)	AITL—1 (12.5) Angiocentric ^a^—3 (37.5) Unspecified—4 (50)	AITL—1 (4) Angiocentric ^a^—3 (21) Unspecified—4 (4)	NR	NR	NR	NR	^a^ Reclassified as ENKTL
Mak et al. [57]	153	12 (7.8)	PTCL, NOS—4 (44) ^a^ ALK+ ALCL—2 (22) ^a^ ALK- ALCL—2 (22) ^a^	PTCL, NOS—4 (5) ^a^ ALK+ ALCL—2 (17) ^a^ ALK- ALCL—2 (8) ^a^	NR	NR	NR	NR	^a^ Data reported only for patients with CNS disease at first relapse (*n* = 9)
Yi et al. [14]	228	20 (8.8)^a^	PTCL, NOS—11 (55) AITL—3 (15) ALK+ ALCL—2 (10) ALK- ALCL—2 (10) ALKu ALCL ^a^—1 (5) EATL—1 (5)	PTCL, NOS—11 (8.5) AITL—3 (5.8) ALK+ ALCL—2 (18.2) ALK- ALCL—2 (16.6) ALKu ALCL—1 (11.1) EATL—1 (12.5)	Leptomeningeal—14 (70) Parenchymal—5 (25) Mixed—1 (5)	Isolated CNS—2 (10) CNS + systemic—18 (90)	6.1	3	^a^ 2 patients had CNS disease at diagnosis
Ellin et al. [15]	625	28 (4.5)	PTCL, NOS—15 (53.6) AITL—3 (10.7) ALK+ ALCL—3 (10.7) ALK- ALCL—2 (7.1) ALKu ALCL—1 (3.6) EATL—4 (14.3)	PTCL, NOS—15 (7) AITL—3 (3) ALK+ ALCL—3 (6) ALK- ALCL—2 (2) ALKu ALCL—1 (3) EATL—4 (7)	Leptomeningeal—18 (64.3) Parenchymal—10 (35.7)	Isolated CNS—11 (52.3) ^a^ CNS + systemic—10 (47.6) ^a^	4.3	1.1	^a^ Reported only for patients with CNS disease at first relapse (*n* = 21)
Gurion et al. [17]	231	15 (6.5)^a^	PTCL, NOS—6 (40) AITL—1 (6.7) ALK- ALCL—1 (6.7) ENKTL—2 (13.3) ATLL—4 (26.7) HSTCL—1 (6.7)	PTCL, NOS—6 (8.2) AITL—1 (2.7) ALK- ALCL—1 (3.6) ENKTL—2 (11.8) ATLL—4 [(23.5) HSTCL—1 (11.1)	NR	Isolated CNS—3 (37.5) ^b^ CNS + systemic 5 (62.5) ^b^	3.4	2.6	^a^ 4 patients had CNS disease prior to first line therapy ^b^ Reported only for patients with CNS disease after first line therapy (*n* = 8)
Chihara et al. [18]	600	13 (2.2)	PTCL, NOS—4 (30.8) AITL—1 (7.7) ALK+ ALCL—4 (30.8) ALK- ALCL—2 (15.4) ENTKL—2 (15.4)	PTCL, NOS—4 (2.3) AITL—1 (0.7) ALK+ ALCL—4 (5.4) ALK- ALCL—2 (1.9) ENTKL—2 (3.7)	Leptomeningeal—13 (100) Parenchymal—0 (0)	NR	6.4	1.5	
Mocikova et al. [19] ^a^	1040	13 (1.3)	PTCL, NOS—10 (76.9) AITL—1 (7.7) ALK- ALCL—1 (7.7) EATL ^b^—1 (7.7)	PTCL, NOS—10 (2.4) AITL—1 (0.9) ALK- ALCL—1 (0.6) EATL ^b^—1 (2.6)	Leptomeningeal—4 (30.8) Parenchymal—6 (46.2) Mixed—3 (23.1)	Isolated CNS—2 (15.4) CNS + systemic—11 (84.6)	0	11 ^c^	^a^ Patients with CNS involvement at initial diagnosis ^b^ EATL and MEITL are separate diagnoses ^c^ OS reported only for patients with CNS + systemic disease at diagnosis
Mocikova et al. [19] ^a^	1040	16 (1.5)	PTCL, NOS—8 (50) ALK+ ALCL—2 (12.5) ALK- ALCL—2 (12.5) AITL—1 (6.25) EATL ^c^—1 (6.25) Other—1 (6.25)	PTCL, NOS—8 (3.4) ^b^ ALK+ ALCL—2 (2.9) ^b^ ALK- ALCL—2 (10.5) ^b^ AITL—1 (1.6) ^b^ EATL ^c^—1 (5) ^b^ Other—1 (0.9) ^b^	Leptomeningeal—6 (37.5) Parenchymal—5 (31.3) Mixed—5 (31.3])	Isolated CNS—4 (25) CNS + systemic—12 (75)	NR	11.8	^a^ Patients with secondary CNS relapse ^b^ Compared within population of patients who experienced relapse (*n* = 509) ^c^ EATL and MEITL are separate diagnoses ^c^ OS from initial diagnosis

^§^ All patients diagnosed with a MTNKL. ^#^ Percent of patients with CNS relapse via histology compared to total patients with CNS relapse. ^$^ Percent of patients with CNS relapse by histology compared to total patients with that histologic diagnosis. ^†^ Median time from initial diagnosis to CNS relapse and/or progression. ^‡^ Median overall survival after CNS relapse. NR: not reported.

## Data Availability

The material supporting the information of this review has been included within this article.
